# Lipids Alterations Associated with Metformin in Healthy Subjects: An Investigation Using Mass Spectrometry Shotgun Approach

**DOI:** 10.3390/ijms231911478

**Published:** 2022-09-29

**Authors:** Lina A. Dahabiyeh, Muhammad Mujammami, Reem H. AlMalki, Tawfiq Arafat, Hicham Benabdelkamel, Assim A. Alfadda, Anas M. Abdel Rahman

**Affiliations:** 1 Department of Pharmaceutical Sciences, School of Pharmacy, The University of Jordan, Amman 11942, Jordan; 2 Endocrinology and Diabetes Unit, Department of Medicine, College of Medicine, King Saud University, Riyadh 12372, Saudi Arabia; 3 University Diabetes Center, King Abdulaziz University Hospital, King Saud University, Riyadh 12372, Saudi Arabia; 4 Department of Botany and Microbiology, College of Science, King Saud University, Riyadh 11451, Saudi Arabia; 5 Metabolomics Section, Department of Clinical Genomics, Center for Genome Medicine, King Faisal Specialist Hospital and Research Centre (KFSHRC), Riyadh 11564, Saudi Arabia; 6 Jordan Center for Pharmaceutical Research, Amman 11195, Jordan; 7 Proteomics Resource Unit, Obesity Research Center, College of Medicine, King Saud University, Riyadh 12372, Saudi Arabia; 8 Department of Biochemistry and Molecular Medicine, College of Medicine, Al Faisal University, Riyadh 11533, Saudi Arabia; 9 Department of Chemistry, Memorial University of Newfoundland, St. John’s, NL A1B 3X7, Canada

**Keywords:** metformin, arachidonic acid, sphingosine-1-phosphate, hydroxyeicosatetraenoic acids (HETE), glycerophospholipid, cancer, T2DM

## Abstract

Metformin is an orally effective insulin-sensitizing drug widely prescribed for treating type 2 diabetes mellitus (T2DM). Metformin has been reported to alter lipid metabolism. However, the molecular mechanisms behind its impact on lipid metabolism remain partially explored and understood. In the current study, mass spectrometry-based lipid profiling was used to investigate the lipidomic changes in the serum of 26 healthy individuals after a single-dose intake of metformin. Samples were analyzed at five-time points: preadministration, before the maximum concentration of metformin (Cmax), Cmax, after Cmax, and 36 h post-administration. A total of 762 molecules were significantly altered between the five-time points. Based on a comparison between baseline level and Cmax, metformin significantly increased and decreased the level of 33 and 192 lipids, respectively (FDR ≤ 0.05 and fold change cutoff of 1.5). The altered lipids are mainly involved in arachidonic acid metabolism, steroid hormone biosynthesis, and glycerophospholipid metabolism. Furthermore, several lipids acted in an opposed or similar manner to metformin levels and included fatty acyls, sterol lipids, glycerolipids, and glycerophospholipids. The significantly altered lipid species pointed to fundamental lipid signaling pathways that could be linked to the pleiotropic effects of metformin in T2DM, insulin resistance, polycystic ovary syndrome, cancer, and cardiovascular diseases.

## 1. Introduction

Metformin, a synthetic dimethyl biguanide, is an orally effective insulin-sensitizing drug widely prescribed for treating type 2 diabetes mellitus (T2DM) as a monotherapy or adjunct therapy to other antihyperglycemic medications [1,2,3,4]. It is a relatively inexpensive and well-tolerated drug with minimal side effects. Gastrointestinal-related side effects such as abdominal pain, bloating, diarrhea, nausea, and vomiting are very common, occurring in 20–30% of patients taking metformin [5]. Lactic acidosis is the most serious side effect associated with metformin; however, it is very rare (incident rate of 1 in 30,000 patients) and can be prevented by inhibiting metformin intake in liver and kidney dysfunction patients [5,6].

The antidiabetic effect of metformin has been related to its ability to inhibit gluconeogenesis by activating adenosine monophosphate (AMP)-activated protein kinase (AMPK), which is considered a central modulator of energy metabolism and glucose homeostasis [7,8,9]. Moreover, metformin reduces intestinal glucose absorption and improves peripheral glucose uptake and insulin sensitivity [10]. Metformin acts on multiple tissues and targets different pathways. In addition to its glucose-lowering effect, it has been reported to exert other beneficial effects, including cardiovascular and neuroprotective effects [5,11,12], weight loss in overweight and obese patients [13], treating metabolic and reproductive abnormalities of polycystic ovary syndrome (PCOS) [14], attenuating tumorigenesis [5,15] and oxidative stress [16,17], and delaying aging [18].

Lipids serve as integral metabolites for diverse biological processes. They are essential structural components of cell membranes and energy storage molecules and play a pivotal role in the pathogenesis of several disorders [19]. The effect of metformin on improving insulin sensitivity is considered a consequence of the changes it induces in lipid metabolism [20]. Clinical studies have demonstrated a lipid-modifying effect of metformin [21,22] by altering fatty acid de novo synthesis, mitochondrial lipid channeling, and β-oxidation [23]. However, the molecular mechanism behind its impact on lipid metabolism remains partially explored and understood.

The expanding development of omics approaches has allowed for a holistic view of various therapeutics’ molecular mechanisms and provided a promising strategy to monitor drugs and innovative compounds [24,25]. Lipids are directly exposed to the biochemical changes linked to pathological processes or drug treatment. Therefore, investigating the lipidome is expected to provide a window to biochemical anomalies associated with the pathophysiology of diseases or drug efficiency and adverse effects [26]. Lipid profiling using mass spectrometry (MS) is a key and advanced analytical strategy capable of identifying and quantifying various lipid species and highlighting the perturbations in lipid metabolism and lipid-mediated signaling processes [27]. Metformin has been reported to induce specific changes in the serum lipidome of women with PCOS [28], and patients with T2DM [29]. However, studying the effect of metformin under pathological conditions might lead to the identification of lipids that are not necessarily altered due to metformin, particularly since most patients undertake metformin as an adjunct therapy to other medications. Therefore, using healthy subjects will identify endogenous metabolites and lipids that are altered specifically due to changes in metformin levels. Recently, we reported the effect of metformin on the biochemical processes in a cohort of healthy volunteers using an MS-based untargeted metabolomics approach where lipid metabolism was among the significantly altered pathways [30]. Therefore, in the current study, an MS-based shotgun approach was used to investigate the alteration in the plasma lipidome (at multiple time points) in healthy subjects in response to acute metformin intake. The above should aid in identifying a potential panel of lipid species disturbed due to metformin administration and thus provide a deeper understanding of the role of metformin in lipid metabolism.

## 2. Results

### 2.1. Clinical and Demographic Data of Study Subjects

The demographic and clinical data of the 26 healthy nondiabetic male participants are presented in Table 1. All lab tests performed during screening and follow-up periods were within the normal range for all subjects, with no significant difference between the two periods. One exception is the level of the liver enzyme alkaline phosphatase (ALP), which was significantly lower in the follow-up period (86 ± 16) compared to the screening period (105 ± 19), Table 1.

### 2.2. Lipid Detection and Data Overview Using Multivariate Analysis

**Figure 1:**
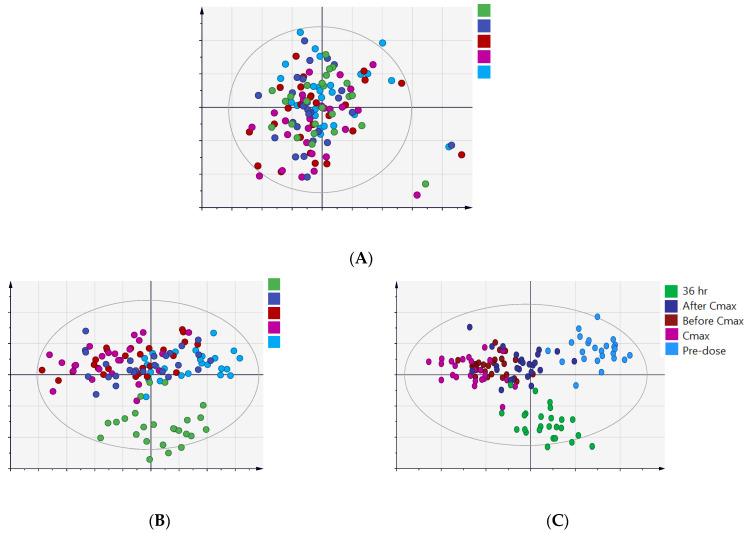
Score plots of the lipidome of serum samples were obtained from 26 healthy subjects after a single dose of metformin at five−time points: predose (baseline level, light blue), 1.5 h before Cmax (red), Cmax (purple), 2 h after Cmax (dark blue), and 36 h post−drug administration (green). (**A**) PCA (R_2_X = 0.68, Q^2^ = 0.42), (**B**) PLS−DA (R_2_X = 0.28, R_2_Y = 0.39, Q2 = 0.15) and (**C**) OPLS−DA (R_2_X = 0.30, R_2_Y = 0.39, Q^2^ = 0.23).

Positive and negative ionization modes collectively detected 9017 mass ions, of which only 2499 mass features remained after applying the missing value filters. The 2499 lipid species were exported for multivariate analysis to overview the data set at the five-time points and identify outliers and possible group clustering and separation (Figure 1). The data were deposited in MetaboLight (accession Number MTBLS2949)

The principal component analysis (PCA) scores plot (Figure 1A) showed an overlap between the datasets with no apparent separation between the five−time points. Clearly, the lipidome of one subject at all time points was outside the confidence interval of the PCA model (Figure 1A). The results of the lab tests for this subject showed an increased level of the two liver enzymes, aspartate transaminase (AST, also known as aspartate aminotransferase) and alanine transaminase (ALT, also known as alanine aminotransferase), during the follow-up period compared to screening tests. It is expected that this subject has a different metabolism than the remaining participants, resulting in a distinguished lipid profile pattern. This is consistent with our previous work where the same subject showed different metabolic profiles at all time points compared to the remaining participants [30]. This unique profile is most probably independent of metformin since even the baseline lipid pattern was different from the remaining individuals. However, this finding might indicate that this subject, due to his different metabolism, was affected by metformin intake, which resulted in the increase in AST and ALT levels. The above specifies that subjects could vary in response to metformin and show the key role of omics in identifying these subjects. For data analysis purposes, this subject was considered an outlier, and his data were removed from the upcoming analyses.

Supervised analysis using partial least-squares discriminative analysis (PLS-DA) and orthogonal PLS-DA (OPLS-DA) showed no clear separation between the five time points, Figure 1B,C, respectively. However, in both models, the predose and 36 h postdose samples were separated from the other time points, with better separation evident in the OPLS-DA score plot (Figure 1C).

### 2.3. The Effect of Metformin on the Lipidome at Four Time Points after Metformin Intake

**Figure 2:**
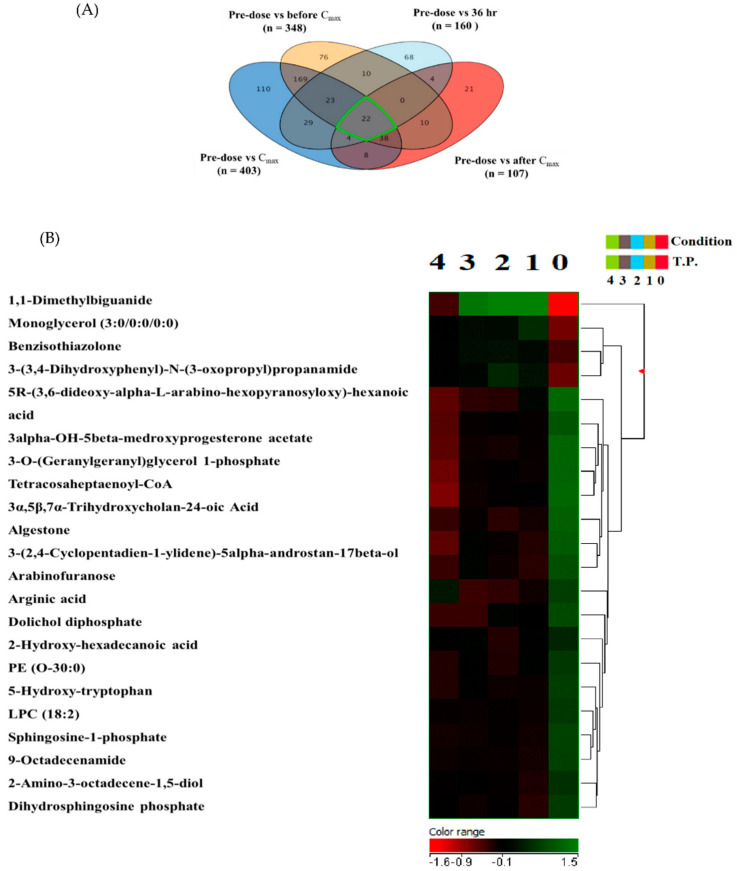
Significantly altered lipids between the post-metformin administration time points compared to the predose sample (baseline level). (**A**) A Venn diagram illustrating the overlap between the post−metformin administration time points (before C_max_, C_max_, after C_max_, 36 h) compared to the predose sample with the number of significantly altered lipids (*n*). A group of 22 features, including metformin, are consistently dysregulated within the five−ime points. Data were analyzed using one-way ANOVA using Tukey’s post hoc analysis. (**B**) Heatmap and hierarchical cluster analysis of 22 features dysregulated between the four−time points after metformin administration and predose sample. Time points 0, 1, 2, 3, and 4 refer to baseline, before C_max_, C_max_, after C_max_ and 36 h post−metformin administration. Metformin is 1,1-Dimethylbiguanide.

The 2499 identified molecular species were statistically evaluated among the five-time points using one-way ANOVA with Tukey’s post hoc analysis. A total of 762 lipids were significantly altered between the five-time points. Statistical comparison between post-metformin administration time points (before the maximum concentration of metformin in the serum (Cmax), Cmax, after Cmax, or 36 h post-administration and predose sample (baseline level) is presented in the Venn diagram shown in Figure 2A. Expectedly, metformin-induced the largest change in the lipidome at its Cmax, with 403 metabolites significantly altered compared to the predose sample. Interestingly, after 36 h of metformin administration, the lipidome did not retain to a level close to the baseline, as 160 lipids were significantly altered between the predose and 36 h samples (Figure 2A). This finding is consistent with the results obtained from the score plot of the OPLS-DA model, where the last two groups showed an apparent separation reflecting different metabolic signatures between the two-time points (Figure 1C). Metformin could not be detected in the circulation after 36 h of intake. Our findings possibly indicate that the effect of metformin on the serum lipidome lasts more than 36 h, and a longer time is needed for the lipidome to return to its baseline level in circulation.

Compared to the baseline level, the Venn diagram revealed a group of 22 features, including metformin, that were commonly dysregulated at the four post-metformin administration time points (Figure 2A,B). Among these altered mass ions are lysosphingolipid (e.g., sphingosine-1-phosphate (S1P)), fatty acids (e.g., 2-hydroxyhexadecanoic acid), glycerophosphates, and glycerophosphocholines. A heat map representing the change in the level of the 22 ion species at the five time points is presented in Figure 2B.

### 2.4. Effect of Metformin on Serum Lipidome at Its Cmax

**Figure 3:**
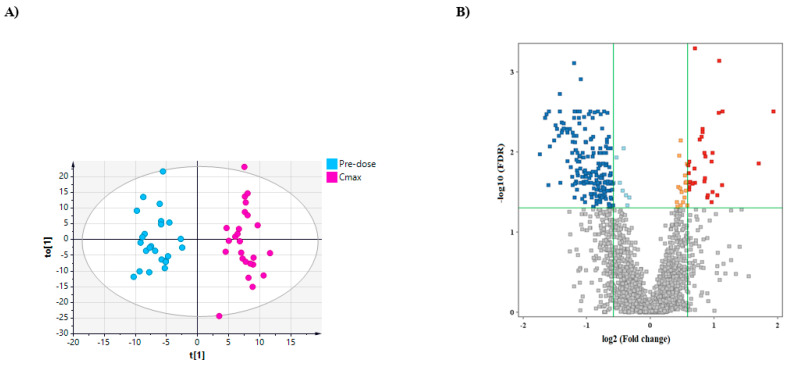
Differentially expressed lipids obtained from 26 subjects after metformin administration based on a binary comparison between baseline level (predose) and C_max_ level. (**A**) OPLS-DA (R2X = 0.26, R2Y = 0.92, Q2 = 0.62) score plot predose (light blue) and at C_max_ (purple). (**B**) Volcano plot (FDR value ≤ 0.05, FC 1.5) of upregulated (red, *n* = 33) and downregulated (blue, *n* = 192) features. Light blue and orange squares refer to lipids that failed to pass fold change cutoffs and were up- and downregulated, respectively. Gray square lipids failed to pass both cutoffs.

Metformin had the most impactful effect on lipid profile or lipidome at its Cmax. The OPLS-DA score plots for serum lipidome at baseline (predose) and Cmax exhibited a clear separation between the two groups, Figure 3A. A volcano plot using a false discovery rate (FDR) value cutoff ≤ 0.05 and a fold change (FC) cutoff of 1.5 revealed a significant dysregulation in the level of 225 features, where the level of 33 and 192 features increased and decreased, respectively, upon metformin administration (Figure 3B). The identity of these compounds together with their changed level are presented in Table S1 in the supplementary material. The altered lipids are involved in various biological processes, including arachidonic acid (AA) metabolism (e.g., 8-hydroxyeicosatetraenoic acid (8-HETE), prostaglandinF2α (PGF2α)), steroid hormone biosynthesis (e.g., androstenedione), and glycerophospholipid metabolism. Furthermore, metformin significantly perturbed the level of bioactive lipid mediators with vital structural and signaling functions such as the sphingolipid, S1P, Table S1.

### 2.5. Metformin-Dependent Lipids

**Figure 4:**
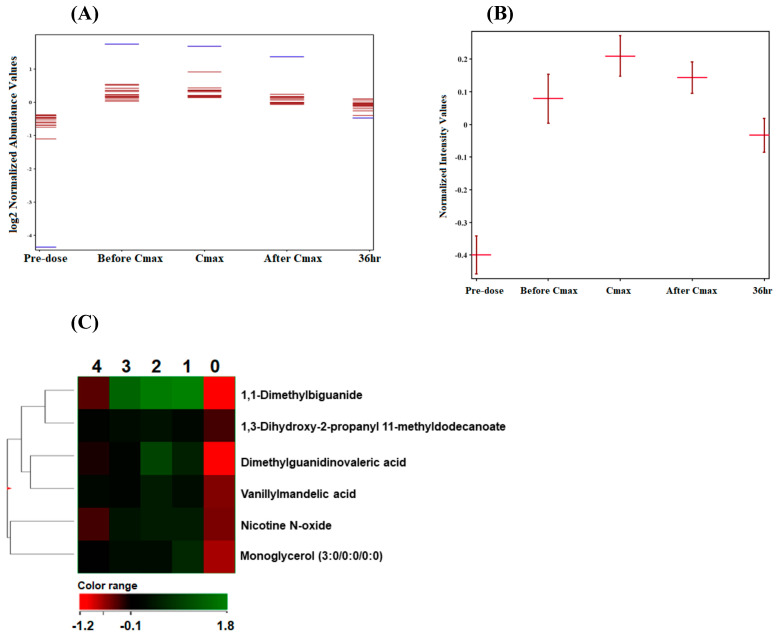
(**A**) Metformin-dependent lipids and metabolites with similar kinetics to metformin. (**B**) Example of the metformin dependent lipid, 1,3-Dihydroxy-2-propanyl 11-methyldodecanoate, showing similar change in its level as metformin. (**C**) Hierarchal clustering (HAC) and heatmap analysis of five metformin-dependent biomolecules showing similar changes in their level to metformin. Time points 0, 1, 2, 3, and 4 refer to predose, before C_max_, C_max_, after C_max_ and 36 h post-metformin administration. Metformin is 1,1-Dimethylbiguanide.

**Figure 5:**
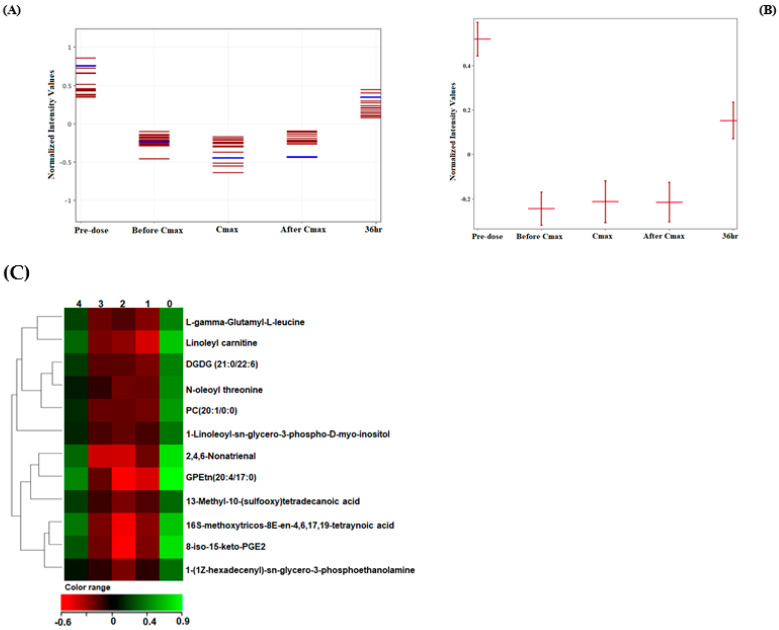
(**A**) Metformin-dependent lipids and metabolites with opposite kinetics to metformin. (**B**) Example of the metformin-dependent lipid, PC (20:1/0:0), showing opposite change in its level compared to metformin. (**C**) Hierarchal clustering (HAC) and heatmap analysis of 12 metformin−dependent biomolecules showing an opposed change in their level compared to metformin. Time points 0, 1, 2, 3, and 4 refer to predose, before Cmax, Cmax, after Cmax, and 36 h post−metformin administration.

The level of the 225 differentially altered metabolites (Section 2.4) was compared between the five time points to identify lipids with a similar or opposite change in their level relative to the metformin pattern (metformin-dependent lipids). The Pearson similarity test revealed that five metabolites (excluding metformin) displayed a similar change in their level compared to metformin, among which are monoglycerols (Figure 4). A larger number of lipids acted in an opposed manner to metformin levels, as presented in Figure 5 and Table 2.

Lipids with opposed levels to metformin patterns included fatty acyls (fatty esters, fatty acids, and eicosanoids), sterol lipids, glycerolipids, and glycerophospholipids (glycerophosphocholines, glycerophosphoinositols).

Heatmaps of metformin-dependent lipids and their levels as similar or opposed to metformin patterns at the five time points are shown in Figure 4C and Figure 5C, respectively.

## 3. Discussion

Metformin is an orally effective insulin-sensitizing biguanide widely prescribed for treating T2DM. Moreover, metformin possesses effective roles in cancer, PCOS, dyslipidemia, and obesity. Using the MS-based metabolomics approach, we previously reported a significant alteration in the lipid metabolism pathway induced by metformin in healthy subjects [30]. Although the effect of metformin on improving insulin sensitivity is considered a consequence of its impact on lipid metabolism, little is known about this effect. Therefore, we used a shotgun lipidomics approach on serum samples from the same healthy subjects to further investigate the role of metformin in lipid metabolism and pathways. Normally, for metformin dosing, the starting dose is 500 mg, then the maintenance will be 850–2550 mg/day for diabetic patients. Herein, the study included healthy subjects as part of a bioequivalent study to monitor the initial and primary metabolic effect of metformin in the dynamic range (<36 h), not at the therapeutic (steady state). Therefore, a single dose of 500 mg of metformin was used.

**Figure 6:**
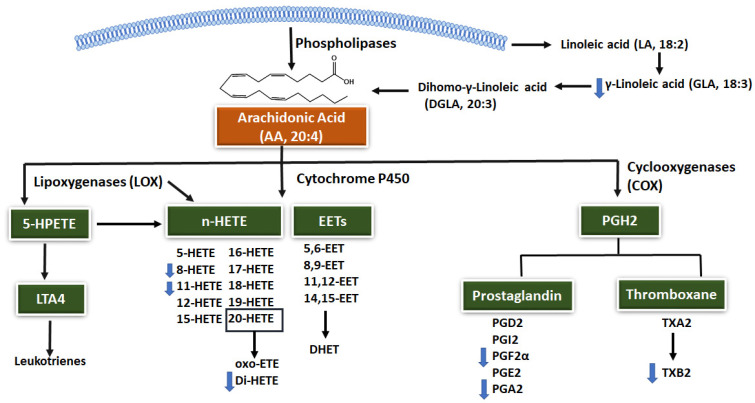
Overview of the arachidonic acid (AA) metabolism pathways. Phospholipases are responsible for releasing AA from membrane-bound phospholipids. AA can also be generated from dihomo-γ-linoleic acid. AA will be metabolized by three pathways: cyclooxygenase (COX), cytochrome P450, and lipoxygenase. PG: prostaglandin, TX: thromboxane, EET: epoxyeicosatrienoic acids, HETE: hydroxyeicosatetraenoic acid, DHETs: dihydroxyeicosatrienoic, LT: leukotrienes. Blue arrows indicate downregulated lipids after metformin administration (based on a comparison between baseline level (predose) and C_max_ level.

Our findings revealed that metformin induced a significant dysregulation in AA metabolism, evident by the downregulation in the level of several lipids involved in this pathway (Figure 6). AA is an important polyunsaturated fatty acid (PUFAs) formed endogenously from its essential fatty acid precursor, linoleic acid (LA 18:2n-6). LA is metabolized to gamma-linolenic acid (GLA), which will be used to generate dihomogamma-linolenic acid (DGLA) and finally AA [31]. However, most of the AA is released from membrane-bound phospholipids by the action of phospholipase enzymes [32], (Figure 6). AA will be further metabolized via three different enzyme pathways: cyclooxygenases (COXs), lipoxygenases (LOXs), and cytochrome P450 enzymes to generate an assortment of biologically active fatty acid mediators, as shown in Figure 6.

The well-known multiple effects of metformin might be linked to its effect on AA metabolism. The AA metabolism pathway provides a spectrum of substrates essential for several key signaling pathways modulating insulin secretion [31]. Moreover, AA and its derivatives activate separate, sometimes overlapping pathways, and thus are considered paramount in several key physiological processes, including inflammation, cardiovascular biology, diabetes, carcinogenesis, and human fertility [31,33]. In the current work, metformin alterations in AA metabolism were manifested in the form of a reduction in the level of GLA and various prostanoids produced via the COX pathway, including PGF2α, PGA2, and TXB2, and eicosanoids produced via the cytochrome P450 and the LOX pathways including 8-HETE and 11-HETE (Figure 6).

The two prostanoids, PGI2 and TXA2 (hydrolyzed toTXB2), are the major platelet aggregation inhibitor and promotors, respectively, that maintain vascular homeostasis and platelet aggregation. Increased levels of TXA2, and consequently TXB2, can contribute to the development of various thrombotic diseases [34]. The level of serum TXB2 is used to reflect the maximum platelet production of TXA2 [35]. T2DM is a well-known risk factor for cardiovascular disease, and higher TXB platelet biosynthesis was reported in patients with T2DM [36,37]. Moreover, T2DM is associated with greater production of 8-iso-prostaglandin PGF2α [38]. PGF2α increases reactive oxygen species (ROS), induces a hypertrophic effect [39], and activates platelet aggregation [38]. The lower level of TXB2 and PGF2α detected herein as of metformin administration indicates that metformin might exert its cardiovascular protective effect by affecting platelet aggregation and decreasing oxidative stress. Our findings are consistent with a previous study where metformin was associated with a significant decrease in 11-dehydro-TXB2 and 8-iso-PGF2α in newly diagnosed T2DM patients [40].

Metformin has also decreased the level of eicosanoids, as reflected by the significant reduction in the level of the two hydroxyeicosatetraenoic acids (HETEs), 8-HETE and 11-HETE. Eicosanoids are much appreciated for their roles in various pathological conditions, including inflammation, free radical generation, metabolic syndrome, and cancer [41,42]. Moreover, HETEs are involved in the development and progression of different solid tumors [43]. The serum concentrations of mid-chain HETE, including 8- and 11-HETE, were elevated in patients with advanced prostate cancer [44], and 11-HETE was also significantly higher in patients with colon hyperplastic polyps and adenomas compared to those with no polyps [45]. Additionally, the level of 11-HETE could be linked to the pathophysiology of diabetes and cardiovascular diseases. A high level of circulating 11-HETE is considered a marker of lipid peroxidation and indicative of oxidative stress conditions and increased ROS [45,46]. All of the above contribute to the peripheral vascular resistance and endothelial dysfunction seen in hypertension and diabetes, respectively [31,36]. Given our findings, metformin may contribute to its reported effect in preventing cancer and tumor progression, protecting against coronary heart diseases, and improving insulin sensitivity by lowering mid-chain HETEs, particularly 11-HETE. To the best of our knowledge, this is the first study to report the effect of metformin in 11-HETE. It is worth noting that 20-HETE remains the best-studied lipid of the HETEs family [34,47] while still, limited literature is available on the physiological role of 11-HETE. This urges the need to investigate further the role of mid-chain HETEs in serious conditions such as T2DM, cancer, and cardiovascular diseases.

In the current work, metformin significantly decreased the level of the bioactive signaling lipid S1P generated by converting ceramide to sphingosine. S1P is involved in many cellular and physiological processes, such as inflammation, immunity and cell survival, proliferation, and migration [48]. Several studies have demonstrated that S1P signaling is closely linked to cancer progression and tumor growth [49,50]. Moreover, growing evidence has highlighted S1P signaling as a potential cancer therapeutic approach by selectively targeting S1P receptors or reducing the levels of S1P itself [49,50]. Considerable effort has been expended in clarifying the beneficial role of metformin in cancer. Our finding suggests that one mechanism by which metformin inhibits tumor growth could be by altering S1P signaling and reducing S1P levels. In line with our work, significantly reduced serum S1P levels have been reported in ovarian cancer patients taking metformin [51]. The same study suggested sphingolipid signaling as a metabolic target of metformin in cancer [51]. Noteworthy, S1P levels have also been associated with the development of obesity, insulin resistance, and T2D [52]. However, this link remains discrepant, and more work is still needed to understand the role of S1P in the development of diabetes.

Growing evidence has underlined the importance of acylcarnitines in various physiological processes such as fatty acid oxidation, energy homeostasis, and insulin secretion and sensitivity regulation, and proliferation of cancer cells [53,54,55]. Herein, metformin disturbed the acylcarnitine pool (mainly long- and short-chain), which could also contribute to its influence on dyslipidemia and T2DM. In addition, the decrease in the level of the two steroidal hormones androstenedione and hydroxyprogesterone induced by metformin can be added to its effect in reducing insulin resistance in PCOS, as both hormones play roles in the pathology of PCOS [28,56,57].

Our results revealed a few lipids that acted similarly to metformin levels, among which are monoglycerol and digalactosyldiacylglycerol (DGDG), respectively (Table 2). The level of these metformin-dependent lipids was increased/decreased upon metformin administration and reserved to baseline level at 36 h postdose. Monoglycerols (monoglycerides) and DGDG are part of the glycerolipids category of lipids structurally characterized by a glycerol backbone linked to fatty acids. Glycerolipids participate in lipid/glucose homeostasis, interlinked with a central process called the glycerolipid/free fatty acid (GL/FFA) cycle [58]. The GL/FFA cycle has two arms: the anabolic arm, to control fatty acid esterification to form triacylglycerol (lipogenesis); and the catabolic arm, to control glycerol release and FFA (lipolysis). Therefore, it is obvious that any perturbations in GL/FFA cycle will result in various illnesses, including T2DM, dyslipidemia, obesity, and insulin resistance [58].

High triacylglycerol and FFA have long contributed to obesity, insulin resistance, and macrovascular diseases. Recent studies revealed that phospholipid alterations might also play a role in the pathological process of metabolic disorders [59]. Phospholipids are divided into two categories: sphingolipids, with a backbone of a sphingosine base; and glycerophospholipids, with a backbone of hydrophobic fatty acid (tails) connected to a hydrophilic phosphate group (head). Modifying the head of the glycerophospholipid with organic molecules such as choline, ethanolamine, or inositol results in the formation of phosphatidylcholine, phosphatidylethanolamine, or phosphatidylinositol, respectively [60]. Phosphatidylcholine and phosphatidylethanolamine are the most abundant phospholipids in the plasma membrane. Therefore, most clinical studies have investigated the relationship between these two glycerophospholipids’ levels, fatty acid composition, and insulin sensitivity. Phosphatidylcholine and phosphatidylethanolamine have been suggested as critical modulators of insulin sensitivity in several ways, altering the fatty acid composition of glycerophospholipids [59]. In a recent large cohort of Chinese individuals, eight plasma glycerophospholipids, especially phosphatidylcholine, were positively associated with the incidence of diabetes [61]. Moreover, glycerophospholipids are associated with the pathophysiology of PCOS [62], and enzymes involved in the glycerophospholipid pathway have been suggested as promising anticancer therapeutic targets [63]. Our results showed that metformin acutely induced a decrease in the level of some glycerophospholipids (e.g., phosphatidylcholine and phosphatidylethanolamine) in an opposed manner to its pattern at the five time points. In line with our data, the level of eight glycerophospholipids declined after 12 weeks of metformin treatment in PCOS patients [28].

Metformin has been reported to have body-weight-lowering effects. Many studies support that metformin can promote weight loss in overweight or obese patients [64,65,66]. Metformin therapy during gestation reduces weight gain and visceral white adipose tissue mass. It offers plenty of effects suitable for inhibiting obesity’s key mechanisms in visceral white adipose tissue, such as inflammation, oxidative stress, and tissue dysfunction [67]. A previous animal study found that metformin has a dual effect on the differentiation of 3T3-L1 preadipocyte cells by promoting or suppressing adipogenesis and that lower concentrations of metformin induce 3T3-L1 preadipocyte differentiation. In comparison, higher concentrations of metformin inhibit adipogenesis [68]. The rat adipocytes culture in a high glucose concentration promoted the basal rate of glycerol release and significantly enhanced the lipolytic action stimulated by either TNF-α or isoproterenol. Metformin inhibits basal lipolysis stimulated by high glucose. It suppresses the high-glucose-enhanced lipolysis response to TNF-α or isoproterenol, reducing free fatty acid concentration and thus improving insulin sensitivity in obese patients and the hyperglycemic conditions of T2DM [69].

It is worth mentioning that literature has pointed to the effect of metformin on clinical lipid profile (e.g., HDL, LDL, triglyceride); however, this effect is pronounced after long-term therapy (>3–6 months) and mainly in diabetic patients [21,70]. Therefore, the effect of metformin on lipid profile (e.g., HDL, LDL, triglyceride) was not measured herein, as only a single dose of metformin was given to healthy subjects, and its effect on the serum lipidome was monitored for 36 h. The association identified herein between several lipid molecular species and metformin suggests that the pleiotropic effect of metformin could be linked to its effect in lipid metabolism and lipid signaling pathways. The alteration in the lipid level was induced acutely after a single dose of metformin. Further investigation is warranted to gain better insights into the functional significance of the current findings under chronic conditions and to validate the potential of metformin-dependent lipids to act as biomarkers to monitor the pharmacological effects of metformin.

## 4. Materials and Methods

### 4.1. Subject Recruitment and Study Design

The detailed study design was published in our recent work [30]. Briefly, 26 healthy male subjects aged 18–50 years were enrolled in the study after obtaining ethical approval (IRB-01-R02) and written informed consent. Subject recruitment and blood sample collection were conducted at Jordan Center for Pharmaceutical Research, Amman, Jordan. Each participant received a single oral dose of 500 mg metformin hydrochloride film-coated tablet under standard fed conditions [30]. For each subject, blood samples were collected at five time points; pre-metformin administration, 1.5 h before the Cmax, Cmax, 2 h after Cmax, and 36 h post-metformin administration, to provide a comprehensive view of the changes in the lipid pattern induced by metformin. Participants’ different clinical data were obtained as specified in our previous study [30]. This included physical examination and measurements of blood pressure, heart rate, blood glucose levels, and HbA1c value. Details on examinations performed during screening and follow-up periods are included in our previous work [30].

### 4.2. Liquid Chromatography–Mass Spectrometry (LC-MS/MS) Lipid Profiling

A total of 130 serum samples (representing five time-point serum samples for each participant) were analyzed using liquid chromatography–high resolution mass spectrometry (LC-MS/MS). Initially, metabolites and lipids were extracted as previously described [71]. Lipid profiling was carried out using QExactive MS coupled with Ultimate 3000 LC (Thermo Fisher Scientific, Santa Clara, CA, USA). Mass ions were separated using an ACQUITY UPLC HSS T3 column (Cat#186003539) (Waters, Milford, MA, USA) and a binary mobile phase composed of 0.05% formic acid (Cat# F0507, Sigma, Burlington, MA, USA) as solvent A and acetonitrile (Cat#: 34851, Sigma) as solvent B. Gradient elution was applied over 16 min at 300 µL/min flow rate. MS spectra were acquired under positive and negative electrospray ionization modes (ESI+, ESI−) with 25,000 enhanced mass resolution in full MS scan (*m*/*z* 50–1500) using data-dependent MS/MS (dd MS/MS) scan mode. Chromatographic and MS parameters were kept as described previously [71].

### 4.3. Data Processing and Lipids Identification

The acquired MS raw data were processed using the Progenesis QI v3.0 software from Waters (Waters Technologies, Milford, MA, USA). A standard pipeline for data processing was followed, starting with peak alignment based on the m/z value and the ion signals’ retention time, peak picking, and then signal filtering based on the peak quality. Features detected in at least 50% of the samples were retained for further analyses. The identity of lipid molecules was depicted mainly by ChemSpider tool on lipid species. The precursor mass and theoretical MS/MS fragmentation tolerance values were set to a 5 ppm mass window and filtered by elemental composition and an isotope similarity score of 80%. Additionally, lipids were identified by matching the MS data obtained with free databases: the Human Metabolome Database (www.hmdb.ca, accessed on 11 November 2021) and LipidMaps (https://www.lipidmaps.org, accessed on 10 November 2021); then LipidBlast, a computer-generated MS/MS database produced by the Metabolomics Fiehn Lab [72], and METLIN (www.metlin.scripps.edu, accessed on 15 November 2021).

### 4.4. Statistical Analysis

Multivariate statistical analysis was performed using SIMCAP+14 (Umetrics AB, Umeå, Sweden). The imported datasets (mass ions with their normalized abundances) were Pareto-scaled, log-transformed, and then used to generate PCA, PLS-DA, and OPLS-DA models. PLS-DA and OPLS-DA models created were evaluated using the fitness-of-model (R2Y) and predictive ability (Q2) values [73]. The model is considered robust when R2Y values are close to 1, and Q2 values are more than 0.5 [73].

Univariate analysis was performed using Mass Profiler Professional v15.0 (MPP) Software (Agilent Inc., Santa Clara, CA, USA). The total sample median was used to normalize the signal and ensure normal distribution. One-way analysis of variance (ANOVA) with Tukey’s post hoc analysis was performed among the five-time points with a significant value of less than 0.05 for the FDR corrected *p*-value. Volcano plot representation was used to identify significantly altered mass features based on an FC cutoff of 1.5 and FDR < 0.05. Venn diagrams and the Pearson similarity test were developed using MPP Software v15.0 (Agilent Inc., Santa Clara, CA, USA) [74]. Heatmap analysis for altered features was performed using the distance measure Pearson. According to the Pearson similarity test, significantly altered lipid species showing similar/adverse kinetics to metformin were classified according to LipidMaps (https://www.lipidmaps.org, accessed on 15 November 2021).

## 5. Conclusions

MS-based lipid profiling for serum samples collected at different time points was applied to unravel the effect of metformin on lipid metabolism in healthy subjects. Our findings revealed that metformin significantly altered AA metabolism by decreasing the level of several prostanoids, eicosanoids, and S1P signaling pathways by suppressing the level of S1P. Moreover, some glycerolipids and glycerophospholipids showed a change in their level as opposed to the metformin pattern. The altered lipids and metformin-dependent lipids could be linked to the well-known favorable effects of metformin in T2DM, insulin resistance, PCOS, cancer, and cardiovascular diseases. A more comprehensive understanding of the mechanisms by which metformin is affecting the S1P signaling pathway remains to be explored in future studies.

One limitation of the study is the absence of samples from females due to the restricted conditions followed in the study design to limit the variations. Subjects lived in a controlled environment (e.g., diet, health status, etc.) and were under clinical monitoring in the center during the metformin intake and the follow-up period, which largely limited the participation of females. Therefore, investigating the level of metformin-dependent lipids in females newly diagnosed with PCOS before and after treatment with metformin remains crucial to validate the role of the discovered potential biomarkers in PCOS. Moreover, further research is warranted to highlight the effect of metformin in the identified dysregulated lipid pattern under pathological conditions, including diabetes. Once validated, metformin-dependent lipids might be used as promising biomarkers to monitor the effect of metformin and predict the potential effect of other therapeutics and chemical compounds.

## Figures and Tables

**Table 1:** Clinical and demographic data of recruited subjects (*n* = 26 male) during screening and follow-up periods.

Clinical and Demographic Data	Mean ± SD.	
	Screening	Follow-Up
Body mass index (BMI, kg/m^2^), (range)	25 ± 3.8, (19.2–29.3)	-
Age (years)	31 ± 9.2	-
Blood pressure (mm Hg)	≤120/80	≤120/80
Heart rate (beat/minute)	69.6 ± 4	71.9 ± 6.5
Glucose (mmole/L)	5.45 ± 0.44	5.08 ± 0.54
Urea (mmole/L)	10.64 ± 2.11	11.17 ± 2.39
Creatinine (umole/L)	91.96 ± 12.38	95.45 ± 9.73
Sodium (mEq/L)	143.2 ± 2.7	143 ± 1.9
Potassium (mEq/L)	4.3 ± 0.2	4.2 ± 0.17
AST (IU/L)	21.2 ± 6.9	26 ± 17.0
ALT (IU/L)	26.2 ± 11.1	30 ± 34.0
ALP ^a^ (IU/L)	105 ± 19.0	86 ± 16.0 *
Total protein (g/dL)	7.4 ± 0.5	7.7 ± 0.5
Total bilirubin (umole/L)	8.04 ± 2.40	8.55 ± 0.5
HbA1c (%)	5.2 ± 0.23	-

Data are presented as mean ± standard deviation. Results of hematology and differential leucocytes count are presented in our previous publication [30]. Examinations during the screening period were performed up to 14 days of pre-metformin administration, while follow-up examinations for the same subjects were performed up to 7 days post-metformin administration. All lab tests were within the normal range with no significant difference between screening and follow-up periods except ALK. ^a^ Lab test values were significantly different between screening and follow-up periods (independent *t*-test, * *p*-value ≤ 0.05). AST: aspartate transaminase, ALT: alanine transaminase, ALP: Alkaline phosphatase.

**Table 2:** Metformin-dependent lipids and their classification.

Category	Common Name	Systematic Name	Abbrev	Main Class	Sub Class	Level Change Compared to Metformin
**Fatty Acyls**	2E,4E,6Z-Nonatrienal	2E,4E,6Z-Nonatrienal	FAL 9:3	Fatty aldehydes	N.A.	Opposite
	O-linoleoylcarnitine	3-[(9Z,12Z)-octadeca-9,12-dienoyloxy]-4-(trimethylazaniumyl) butanoate	CAR 18:2	Fatty esters	Fatty acids and conjugates Unsaturated fatty acids	Opposite
				Fatty acyl carnitines		
	Sufac#1	13-methyl-10-(sulfooxy)tetradecanoic acid		Fatty acids and conjugates	Branched fatty acids	Opposite
	Carduusyne C	16S-methoxytricos-8E-en-4,6,17,19-tetraynoic acid	FA 24:9;O	Fatty acids and conjugates	Methoxy fatty acids	Opposite
	8-iso-15-keto-PGE2	9,15-dioxo-11R-hydroxy-5Z,13E-prostadienoic acid-cyclo [8S,12R]	FA 20:5;O3	Eicosanoids	Isoprostanes	Opposite
**Glycerophospholipids**	PC(14:0/20:4(5Z,8Z,11Z,14Z))	1-tetradecanoyl-2-(5Z,8Z,11Z,14Z-eicosatetraenoyl)-sn-glycero-3-phosphocholine	PC 34:4	Glycerophosphocholines	Diacylglycerophosphocholines	Opposite
	PC(20:1(9Z)/0:0)	1-(9Z-eicosenoyl)-sn-glycero-3-phosphocholine	LPC 20:1	Glycerophosphocholines	Monoacylglycerophosphocholines	Opposite
	PE(P-16:0/0:0)	1-(1Z-hexadecenyl)-sn-glycero-3-phosphoethanolamine	LPE O-16:1	Glycerophosphoethanolamines	1Z-alkenylglycerophosphoethanolamines	Opposite
	PI(18:2(9Z,12Z)/0:0)	1-(9Z,12Z-octadecadienoyl)-glycero-3-phospho-(1′-myo-inositol)	LPI 18:2	Glycerophosphoinositols	Monoacylglycerophosphoinositols	Opposite

Lipids’ systematic names and classes were obtained from LIPID MAPS^®^ Structure Database.

## Data Availability

The raw data of this study were deposited to MetaboLights, and can be accessed through this link https://www.ebi.ac.uk/metabolights/editor/www.ebi.ac.uk/metabolights/MTBLS2949, accessed on 11 November 2021.

## Supplementary Materials

The following supporting information can be downloaded at: https://www.mdpi.com/article/10.3390/ijms231911478/s1.

## References

[1] Buse J.B., Wexler D.J., Tsapas A., Rossing P., Mingrone G., Mathieu C., D’Alessio D.A., Davies M.J. (2020). 2019 Update to: Management of Hyperglycemia in Type 2 Diabetes, 2018. A Consensus Report by the American Diabetes Association (ADA) and the European Association for the Study of Diabetes (EASD). Diabetes Care.

[2] Davies M.J., D’Alessio D.A., Fradkin J., Kernan W.N., Mathieu C., Mingrone G., Rossing P., Tsapas A., Wexler D.J., Buse J.B. (2018). Management of Hyperglycemia in Type 2 Diabetes, 2018. A Consensus Report by the American Diabetes Association (ADA) and the European Association for the Study of Diabetes (EASD). Diabetes Care.

[3] Rojas L.B.A., Gomes M.B. (2013). Metformin: An old but still the best treatment for type 2 diabetes. Diabetol. Metab. Syndr..

[4] Baker C., Retzik-Stahr C., Singh V., Plomondon R., Anderson V., Rasouli N. (2021). Should metformin remain the first-line therapy for treatment of type 2 diabetes?. Ther. Adv. Endocrinol. Metab..

[5] Wang Y.-W., He S.-J., Feng X., Cheng J., Luo Y.-T., Tian L., Huang Q. (2017). Metformin: A review of its potential indications. Drug Des. Dev. Ther..

[6] Huang W., Castelino R.L., Peterson G.M. (2017). Lactate Levels with Chronic Metformin Use: A Narrative Review. Clin. Drug Investig..

[7] Rena G., Hardie D.G., Pearson E.R. (2017). The mechanisms of action of metformin. Diabetologia.

[8] Viollet B., Guigas B., Garcia N.S., Leclerc J., Foretz M., Andreelli F. (2012). Cellular and molecular mechanisms of metformin: An overview. Clin. Sci..

[9] Zhang B.B., Zhou G., Li C. (2009). AMPK: An Emerging Drug Target for Diabetes and the Metabolic Syndrome. Cell Metab..

[10] Grzybowska M., Bober J., Olszewska M. (2011). Metformin—Mechanisms of action and use for the treatment of type 2 diabetes mellitus. Postepy Hig. I Med. Dosw..

[11] Driver C., Bamitale K.D.S., Kazi A., Olla M., Nyane N.A., Owira P.M.O. (2018). Cardioprotective Effects of Metformin. J. Cardiovasc. Pharmacol..

[12] Guo M., Mi J., Jiang Q.-M., Xu J.-M., Tang Y.-Y., Tian G., Wang B. (2014). Metformin may produce antidepressant effects through improvement of cognitive function among depressed patients with diabetes mellitus. Clin. Exp. Pharmacol. Physiol..

[13] Seifarth C., Schehler B., Schneider H.J. (2013). Effectiveness of Metformin on Weight Loss in Non-Diabetic Individuals with Obesity. Exp. Clin. Endocrinol. Diabetes.

[14] Barbieri R.L. (2003). Metformin for the treatment of polycystic ovary syndrome. Obstet. Gynecol..

[15] Yu X., Mao W., Zhai Y., Tong C., Liu M., Ma L., Yu X., Li S. (2016). Anti-tumor activity of metformin: From metabolic and epigenetic perspectives. Oncotarget.

[16] Bonnefont-Rousselot D., Raji B., Walrand S., Gardès-Albert M., Jore D., Legrand A., Peynet J., Vasson M. (2003). An intracellular modulation of free radical production could contribute to the beneficial effects of metformin towards oxidative stress. Metabolism Clin. Exp..

[17] Kane D.A., Anderson E.J., Price J.W., Woodlief T., Lin C.-T., Bikman B.T., Cortright R.N., Neufer P.D. (2010). Metformin selectively attenuates mitochondrial H_2_O_2_ emission without affecting respiratory capacity in skeletal muscle of obese rats. Free Radic. Biol. Med..

[18] Mohammed I., Hollenberg M.D., Ding H., Triggle C.R. (2021). A Critical Review of the Evidence That Metformin Is a Putative Anti-Aging Drug That Enhances Healthspan and Extends Lifespan. Front. Endocrinol..

[19] Ott J., Hiesgen C., Mayer K. (2011). Lipids in critical care medicine. Prostaglandins Leukot. Essent. Fat. Acids.

[20] Pernicova I., Korbonits M. (2014). Metformin—Mode of action and clinical implications for diabetes and cancer. Nat. Rev. Endocrinol..

[21] Lin S.H., Cheng P.C., Tu S.T., Hsu S.R., Cheng Y.C., Liu Y.H. (2018). Effect of metformin monotherapy on serum lipid profile in statin-naïve individuals with newly diagnosed type 2 diabetes mellitus: A cohort study. PeerJ.

[22] Kashi Z., Mahrooz A., Kianmehr A., Alizadeh A. (2016). The Role of Metformin Response in Lipid Metabolism in Patients with Recent-Onset Type 2 Diabetes: HbA1c Level as a Criterion for Designating Patients as Responders or Nonresponders to Metformin. PLoS ONE.

[23] Zabielski P., Hady H.R., Chacinska M., Roszczyc K., Górski J., Blachnio-Zabielska A.U. (2018). The effect of high fat diet and metformin treatment on liver lipids accumulation and their impact on insulin action. Sci. Rep..

[24] Dahabiyeh L.A., Malkawi A.K., Wang X., Colak D., Mujamammi A.H., Sabi E.M., Li L., Dasouki M., Rahman A.M.A. (2020). Dexamethasone-Induced Perturbations in Tissue Metabolomics Revealed by Chemical Isotope Labeling LC-MS Analysis. Metabolites.

[25] Dahabiyeh L., Mahmoud N., Al-Natour M., Safo L., Kim D.-H., Khalil E., Abu-Dahab R. (2021). Phospholipid-Gold Nanorods Induce Energy Crisis in MCF-7 Cells: Cytotoxicity Evaluation Using LC-MS-Based Metabolomics Approach. Biomolecules.

[26] Stephenson D.J., Hoeferlin L.A., Chalfant C.E. (2017). Lipidomics in translational research and the clinical significance of lipid-based biomarkers. Transl. Res..

[27] Spener F., Lagarde M., Géloên A., Record M. (2003). What is lipidomics?. Eur. J. Lipid Sci. Technol..

[28] Pradas I., Rovira-Llopis S., Naudí A., Bañuls C., Rocha M., Hernandez-Mijares A., Pamplona R., Victor V.M., Jové M. (2019). Metformin induces lipid changes on sphingolipid species and oxidized lipids in polycystic ovary syndrome women. Sci. Rep..

[29] Zhang Y., Hu C., Hong J., Zeng J., Lai S., Lv A., Su Q., Dong Y., Zhou Z., Tang W. (2014). Lipid Profiling Reveals Different Therapeutic Effects of Metformin and Glipizide in Patients with Type 2 Diabetes and Coronary Artery Disease. Diabetes Care.

[30] Dahabiyeh L.A., Mujammami M., Arafat T., Benabdelkamel H., Alfadda A.A., Rahman A.M.A. (2021). A Metabolic Pattern in Healthy Subjects Given a Single Dose of Metformin: A Metabolomics Approach. Front. Pharmacol..

[31] Das U.N. (2018). Arachidonic acid in health and disease with focus on hypertension and diabetes mellitus: A review. J. Adv. Res..

[32] Innes J.K., Calder P.C. (2018). Omega-6 fatty acids and inflammation. Prostaglandins Leukot. Essent. Fat. Acids.

[33] Szczuko M., Kikut J., Komorniak N., Bilicki J., Celewicz Z., Ziętek M. (2020). The Role of Arachidonic and Linoleic Acid Derivatives in Pathological Pregnancies and the Human Reproduction Process. Int. J. Mol. Sci..

[34] Wang B., Wu L., Chen J., Dong L., Chen C., Wen Z., Hu J., Fleming I., Wang D.W. (2021). Metabolism pathways of arachidonic acids: Mechanisms and potential therapeutic targets. Signal Transduct. Target. Ther..

[35] Szczuko M., Kozioł I., Kotlęga D., Brodowski J., Drozd A. (2021). The Role of Thromboxane in the Course and Treatment of Ischemic Stroke: Review. Int. J. Mol. Sci..

[36] Nusca A., Tuccinardi D., Pieralice S., Giannone S., Carpenito M., Monte L., Watanabe M., Cavallari I., Maddaloni E., Ussia G.P. (2021). Platelet Effects of Anti-diabetic Therapies: New Perspectives in the Management of Patients with Diabetes and Cardiovascular Disease. Front. Pharmacol..

[37] Davì G., Catalano I., Averna M., Notarbartolo A., Strano A., Ciabattoni G., Patrono C. (1990). Thromboxane Biosynthesis and Platelet Function in Type II Diabetes Mellitus. N. Engl. J. Med..

[38] Davi G., Ciabattoni G., Consoli A., Mezzetti A., Falco A., Santarone S., Pennese E., Vitacolonna E., Bucciarelli T., Costantini F. (1999). In vivo formation of 8-iso-prostaglandin F-2 alpha and platelet activation in diabetes mellitus—Effects of improved metabolic control and vitamin E supplementation. Circulation.

[39] Rice K.M., Uddemarri S., Desai D.H., Morrison R.G., Harris R., Wright G.L., Blough E. (2008). PGF2α-associated vascular smooth muscle hypertrophy is ROS dependent and involves the activation of mTOR, p70S6k, and PTEN. Prostaglandins Lipid Mediat..

[40] Formoso G., De Filippis E.A., Michetti N., Di Fulvio P., Pandolfi A., Bucciarelli T., Ciabattoni G., Nicolucci A., Davì G., Consoli A. (2008). Decreasedin vivo oxidative stress and decreased platelet activation following metformin treatment in newly diagnosed type 2 diabetic subjects. Diabetes Metab. Res. Rev..

[41] Dennis E.A., Norris P.C. (2015). Eicosanoid storm in infection and inflammation. Nat. Rev. Immunol..

[42] Wang D., DuBois R.N. (2010). Eicosanoids and cancer. Nat. Rev. Cancer.

[43] Evangelista E.A., Cho C.W., Aliwarga T., Totah R.A. (2020). Expression and Function of Eicosanoid-Producing Cytochrome P450 Enzymes in Solid Tumors. Front. Pharmacol..

[44] Rodríguez-Blanco G., Burgers P.C., Dekker L.J.M., Ijzermans J.J.N., Wildhagen M.F., Schenk-Braat E.A.M., Bangma C.H., Jenster G., Luider T.M. (2014). Serum levels of arachidonic acid metabolites change during prostate cancer progression. Prostate.

[45] Pickens C.A., Yin Z., Sordillo L.M., Fenton J.I. (2019). Arachidonic acid-derived hydroxyeicosatetraenoic acids are positively associated with colon polyps in adult males: A cross-sectional study. Sci. Rep..

[46] Guido D.M., McKenna R., Mathews W.R. (1993). Quantitation of Hydroperoxy-Eicosatetraenoic Acids and Hydroxy-Eicosatetraenoic Acids as Indicators of Lipid Peroxidation Using Gas Chromatography-Mass Spectrometry. Anal. Biochem..

[47] Tsai M.-J., Chang W.-A., Tsai P.-H., Wu C.-Y., Ho Y.-W., Yen M.-C., Lin Y.-S., Kuo P.-L., Hsu Y.-L. (2017). Montelukast Induces Apoptosis-Inducing Factor-Mediated Cell Death of Lung Cancer Cells. Int. J. Mol. Sci..

[48] Grassi S., Mauri L., Prioni S., Cabitta L., Sonnino S., Prinetti A., Giussani P. (2019). Sphingosine 1-Phosphate Receptors and Metabolic Enzymes as Druggable Targets for Brain Diseases. Front. Pharmacol..

[49] Pyne N.J., Pyne S. (2020). Recent advances in the role of sphingosine 1-phosphate in cancer. FEBS Lett..

[50] Wang P., Yuan Y., Lin W., Zhong H., Xu K., Qi X. (2019). Roles of sphingosine-1-phosphate signaling in cancer. Cancer Cell Int..

[51] Hart P.C., Chiyoda T., Liu X., Weigert M., Curtis M., Chiang C.-Y., Loth R., Lastra R., McGregor S.M., Locasale J.W. (2019). SPHK1 Is a Novel Target of Metformin in Ovarian Cancer. Mol. Cancer Res..

[52] Guitton J., Bandet C.L., Mariko M.L., Tan-Chen S., Bourron O., Benomar Y., Hajduch E., Le Stunff H. (2020). Sphingosine-1-Phosphate Metabolism in the Regulation of Obesity/Type 2 Diabetes. Cells.

[53] Reuter S.E., Evans A.M. (2012). Carnitine and Acylcarnitines Pharmacokinetic, Pharmacological and Clinical Aspects. Clin. Pharmacokinet..

[54] Mai M., Tönjes A., Kovacs P., Stumvoll M., Fiedler G.M., Leichtle A.B. (2013). Serum Levels of Acylcarnitines Are Altered in Prediabetic Conditions. PLoS ONE.

[55] Armitage E.G., Southam A.D. (2016). Monitoring cancer prognosis, diagnosis and treatment efficacy using metabolomics and lipidomics. Metabolomics.

[56] O’Reilly M., Taylor A.E., Crabtree N.J., Hughes B.A., Capper F., Crowley R., Stewart P.M., Tomlinson J., Arlt W. (2014). Hyperandrogenemia Predicts Metabolic Phenotype in Polycystic Ovary Syndrome: The Utility of Serum Androstenedione. J. Clin. Endocrinol. Metab..

[57] Maas K.H., Chuan S.S., Cook-Andersen H., Su H.I., Duleba A., Chang R.J. (2015). Relationship Between 17-Hydroxyprogesterone Responses to Human Chorionic Gonadotropin and Markers of Ovarian Follicle Morphology in Women With Polycystic Ovary Syndrome. J. Clin. Endocrinol. Metab..

[58] Prentki M., Madiraju S.R.M. (2012). Glycerolipid/free fatty acid cycle and islet β-cell function in health, obesity and diabetes. Mol. Cell. Endocrinol..

[59] Chang W., Hatch G.M., Wang Y., Yu F., Wang M. (2018). The relationship between phospholipids and insulin resistance: From clinical to experimental studies. J. Cell. Mol. Med..

[60] Hishikawa D., Hashidate T., Shimizu T., Shindou H. (2014). Diversity and function of membrane glycerophospholipids generated by the remodeling pathway in mammalian cells. J. Lipid Res..

[61] Chen S., Zong G., Wu Q., Yun H., Niu Z., Zheng H., Zeng R., Sun L., Lin X. (2022). Associations of plasma glycerophospholipid profile with modifiable lifestyles and incident diabetes in middle-aged and older Chinese. Diabetologia.

[62] Jové M., Pradas I., Naudí A., Rovira-Llopis S., Bañuls C., Rocha M., Portero-Otin M., Hernández-Mijares A., Victor V.M., Pamplona R. (2018). Lipidomics reveals altered biosynthetic pathways of glycerophospholipids and cell signaling as biomarkers of the polycystic ovary syndrome. Oncotarget.

[63] Dolce V., Cappello A.R., Lappano R., Maggiolini M. (2011). Glycerophospholipid Synthesis as a Novel Drug Target Against Cancer. Curr. Mol. Pharmacol..

[64] McDonagh M.S., Selph S., Ozpinar A., Foley C. (2014). Systematic Review of the Benefits and Risks of Metformin in Treating Obesity in Children Aged 18 Years and Younger. JAMA Pediatr..

[65] O’Connor E.A., Evans C.V., Burda B.U., Walsh E.S., Eder M., Lozano P. (2017). Screening for Obesity and Intervention for Weight Management in Children and Adolescents: Evidence Report and Systematic Review for the US Preventive Services Task Force. JAMA.

[66] Graff S.K., Mario F.M., Ziegelmann P., Spritzer P.M. (2016). Effects of orlistat vs. metformin on weight loss-related clinical variables in women with PCOS: Systematic review and meta-analysis. Int. J. Clin. Pract..

[67] Schmitz K., Turnwald E.-M., Kretschmer T., Janoschek R., Bae-Gartz I., Voßbrecher K., Kammerer M.D., Köninger A., Gellhaus A., Handwerk M. (2022). Metformin Prevents Key Mechanisms of Obesity-Related Complications in Visceral White Adipose Tissue of Obese Pregnant Mice. Nutrients.

[68] Chen D., Wang Y., Wu K., Wang X. (2018). Dual Effects of Metformin on Adipogenic Differentiation of 3T3-L1 Preadipocyte in AMPK-Dependent and Independent Manners. Int. J. Mol. Sci..

[69] Ren T., He J., Jiang H., Zu L., Pu S., Guo X., Xu G. (2006). Metformin reduces lipolysis in primary rat adipocytes stimulated by tumor necrosis factor-α or isoproterenol. J. Mol. Endocrinol..

[70] Gillani S.W., Ghayedi N., Roosta P., Seddigh P., Nasiri O. (2021). Effect of Metformin on Lipid Profiles of Type 2 Diabetes Mellitus: A Metaanalysis of Randomized Controlled Trials. J. Pharm. Bioallied Sci..

[71] Aleidi S.M., Dahabiyeh L.A., Gu X., Al Dubayee M., Alshahrani A., Benabdelkamel H., Mujammami M., Li L., Aljada A., Abdel Rahman A.M. (2021). Obesity Connected Metabolic Changes in Type 2 Diabetic Patients Treated With Metformin. Front. Pharmacol..

[72] Kind T., Liu K.-H., Lee D.Y., DeFelice B., Meissen J.K., Fiehn O. (2013). LipidBlast in silico tandem mass spectrometry database for lipid identification. Nat. Methods.

[73] Worley B., Powers R. (2013). Multivariate Analysis in Metabolomics. Curr. Metab..

[74] Gu X.Y., Al Dubayee M., Alshahrani A., Masood A., Benabdelkamel H., Zahra M., Li L., Rahman A.M.A., Aljada A. (2020). Distinctive Metabolomics Patterns Associated with Insulin Resistance and Type 2 Diabetes Mellitus. Front. Mol. Biosci..

